# Improved synthesis and physicochemical characterization of the selective serotonin 2A receptor agonist 25CN-NBOH

**DOI:** 10.3762/bjoc.22.11

**Published:** 2026-01-22

**Authors:** Adrian G Rossebø, Hannah G Kolberg, Anders E Tønder, Louise Kjaerulff, Poul Erik Hansen, Karla A Frydenvang, Jesper Østergaard, Jesper L Kristensen

**Affiliations:** 1 Department of Drug Design and Pharmacology, Faculty of Health and Medical Sciences, University of Copenhagen, Jagtvej 162, 2100 Copenhagen, Denmark; 2 Department of Pharmacy, Faculty of Health and Medical Sciences, University of Copenhagen, Universitetsparken 2, 2100 Copenhagen, Denmark; 3 LEO Foundation Center for Cutaneous Drug Delivery, Department of Pharmacy, Faculty of Health and Medical Sciences, University of Copenhagen, Universitetsparken 2, 2100 Copenhagen, Denmark; 4 Department of Science and Environment, Roskilde University, Universitetsvej 1, 4000 Roskilde, Denmark

**Keywords:** intramolecular hydrogen bond, membrane permeability, solubility, stability, synthesis

## Abstract

25CN-NBOH is a selective serotonin 2A receptor agonist used extensively as a tool compound in preclinical research. Herein, we perform an in-depth characterization of 25CN-NBOH and report key physicochemical properties in the solid state as well as in the solution state, namely stability, ionization, solubility, partition coefficient, and membrane permeability. We show that the hydrochloride salt is a stable and well-defined crystalline compound that is also stable in aqueous solutions at room temperature. Furthermore, we show that an intramolecular hydrogen bond is present in solution, which presumably is a key contributor to the high membrane permeability. Collectively, this physical-chemical profiling of 25CN-NBOH provides guidance to researchers with respect to the physiochemical properties and handling of the compound in various research scenarios.

## Introduction

In recent years, classical psychedelics such as psilocybin and LSD have re-emerged as promising treatments for a variety of indications like depression, anxiety, and substance use disorders [1]. While these compounds have varying pharmacological profiles, they share serotonin 2A receptor (5-HT_2A_R) agonism as a central property, linked to their acute psychoactive effects and therapeutic efficacy [2]. Activation of the 5-HT_2A_R is thought to induce altered states of consciousness and suppress the default-mode network, a process hypothesized to underlie their clinical benefits [3]. However, classic psychedelics lack receptor selectivity, prompting efforts to develop more selective 5-HT_2A_R agonists to study the effects of solely activating this key receptor [4].

25CN-NBOH (**1**) was reported in 2014 as part of a series of ligands in the search for selective 5-HT_2A_R agonists [5]. Subsequently, it has been used and characterized extensively as a tool for investigations into the effects of selective 5-HT_2A_R activation in vitro and in vivo [6]. A review published in 2021 provide an in-depth introduction to the pharmacological properties and various applications [7]. More recent examples include the mapping of the cortical correlates of the so-called head-twitch response in mice [8], enhancement of cognitive flexibility [9], induction of neuroplasticity in cortical neurons [10], and persistent antidepressant-like effects [11].

Given the widespread use of this ligand in basic research, some scattered data on its physicochemical properties are available, but a structured and comprehensive evaluation has not been conducted. The most widely used form of **1** is the corresponding HCl salt (**1·**HCl) that in our experience is a crystalline, nonhygroscopic and free-flowing solid, and aqueous solutions thereof appear stable for weeks at room temperature. To substantiate these qualitative observations, we have investigated the properties of 25CN-NBOH·HCl (**1·**HCl) – both in the solid form and in solution. We also optimized the last step in its synthesis and investigated the structural basis for its high membrane permeability, despite the presence of a free phenolic moiety.

## Results and Discussion

### Synthesis

**Figure 1:**
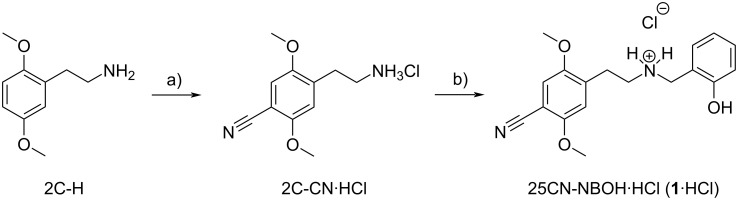
Synthesis of 25CN-NBOH·HCl (**1·**HCl). a) 2C-CN is available in 4 steps from 2C-H [12]: 1) TFAA, TEA, DCM; 2) TiCl_4_, dichloromethyl methyl ether, DCM; 3) NH_2_OH, HCl, EtOH, then Ac_2_O; 4) NaBH_4_, EtOH, then HCl. b) Salicylaldehyde, Et_3_N, 4 Å molecular sieves, EtOH, rt, 1.5 h, then NaBH_4_, 0 °C to rt, 1.2 h, then HCl, 74%.

The synthesis of **1** starts from commercially available 2,5-dimethoxyphenethylamine (2C-H), which can be transformed into the intermediate 2C-CN, (Figure 1). We have previously reported high yielding 4-step procedure for this process, whereas the yield in the final step in the synthesis of **1** was a modest 65% [12].

In the present work, we found that simply adding 4 Å molecular sieves powder during the formation of the intermediate imine gave a much cleaner reaction and increased the yield of **1·**HCl from 2C-CN·HCl to 74%. We suggest that the addition of the molecular sieves facilitates the formation of the required imine intermediate that upon reduction yields the desired product. The free base **1** was purified on normal-phase flash column chromatography, and **1·**HCl was subsequently precipitated, providing a white crystalline solid (vide infra).

### Characterization of 25CN-NBOH·HCl (**1**·HCl) in the solid form

**Figure 2:**
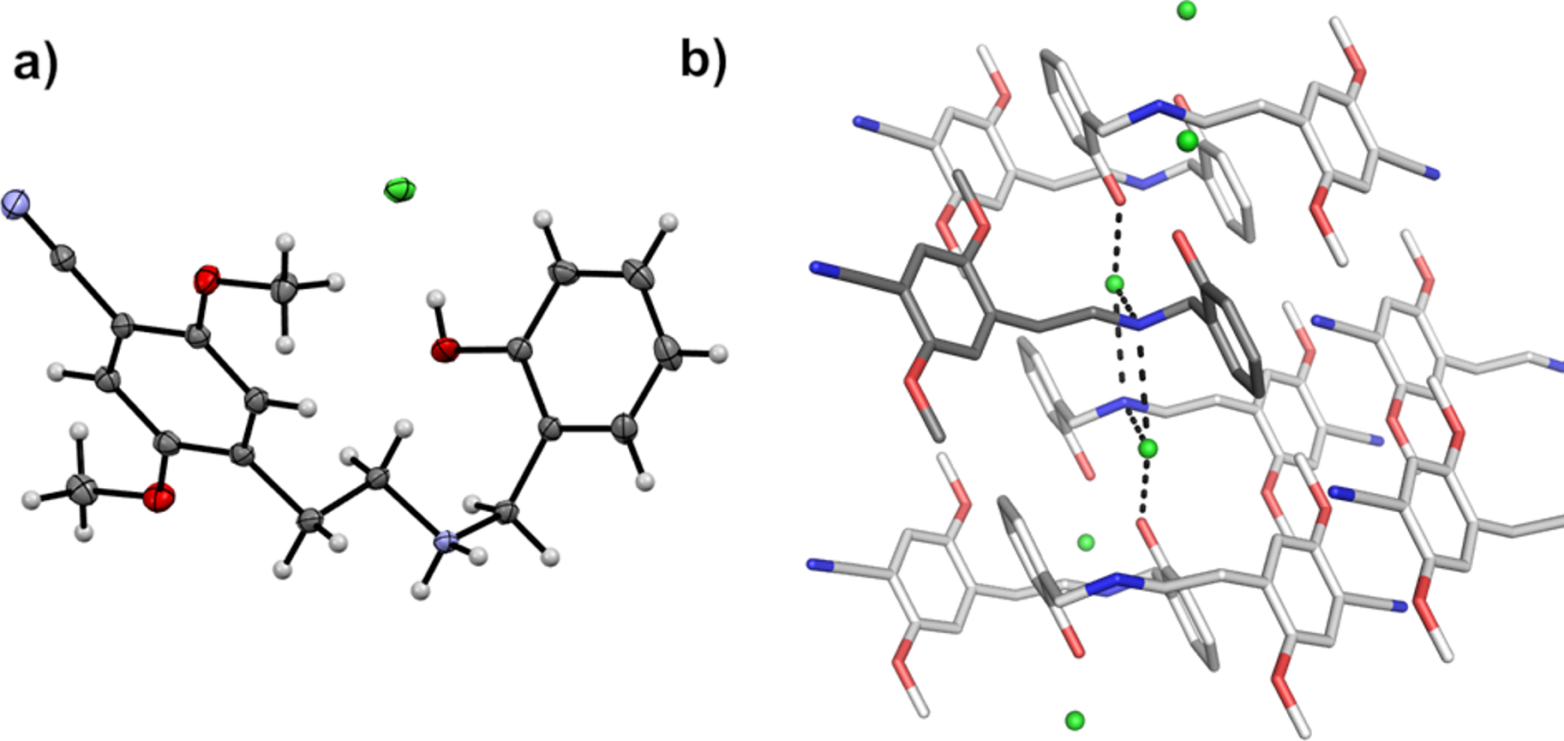
a) Single-crystal X-ray structure of **1·**HCl. Displacement ellipsoids of the nonhydrogen atoms are shown at the 50% probability level. Hydrogen atoms are shown as spheres of arbitrary size. Nitrogen atoms are blue, oxygen atoms red, and chloride ion green. b) The crystal packing reveals alternating layers, a polar layer with hydrogen bonds (black dashed lines), and a hydrophobic layer with π–π-stacking (grey dashed lines).

Crystals of **1·**HCl were grown from an ethanolic solution, and the crystal structure was determined by single-crystal X-ray diffraction (SCXRD, Figure 2).

As seen in Figure 2a, **1·**HCl adopts a twisted U shape and is involved in three hydrogen bonds. Therein, the ammonium N atom interacts with two different chloride ions, and the phenol OH moiety interacts with another chloride ion. The triclinic crystal packing (Figure 2b) reveals alternating layers, a polar layer with the observed hydrogen bonds, and a hydrophobic layer with van-der-Waals interactions and π–π-stacking (distance 4 Å). Notably, the crystal structure shows that there are no water molecules of crystallization present, nor are any other solvent molecules incorporated into the crystal packing.

**Figure 3:**
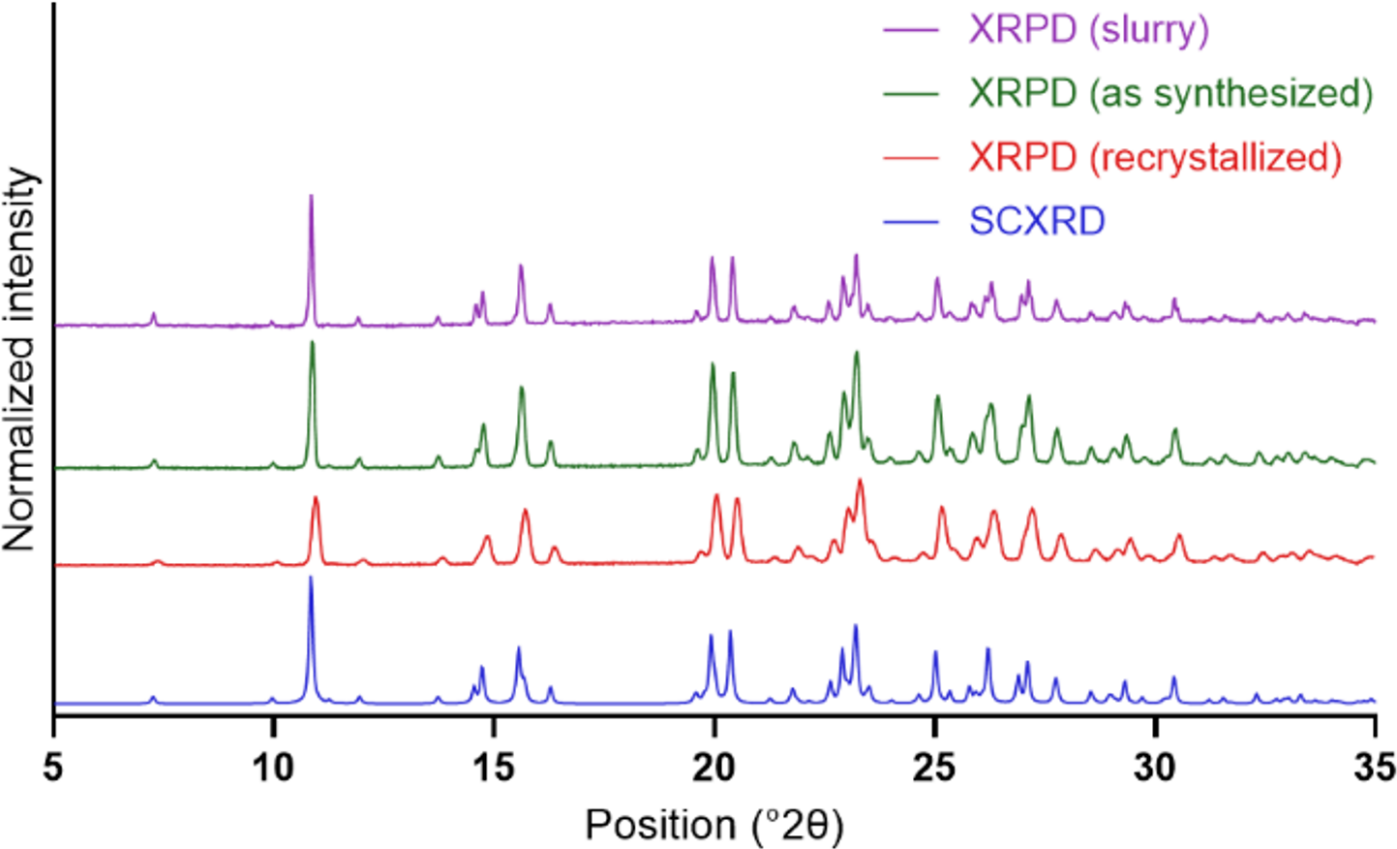
SCXRD and XRPD spectra of different preparations of **1**·HCl. Blue: SCXRD spectrum of **1**·HCl. Red, green, and purple: XRPD spectra of **1**·HCl after recrystallization (red), directly as synthesized (green), and of a slurry obtained by stirring excess solid in ultrapure water for 3 days (purple).

When comparing the diffractogram obtained by SCXRD (Figure 3, green) with that obtained by X-ray powder diffraction (XRPD) of **1·**HCl recrystallized from ethanol (Figure 3, purple), a similar pattern is observed, indicating a high degree of crystallinity. Furthermore, samples of **1·**HCl prepared either by rapid precipitation in organic solvents, as described in the Experimental section (red), or material isolated from a saturated solution of **1·**HCl in ultrapure water (blue), gave comparable diffractograms, indicating the presence of a single polymorph.

**Figure 4:**
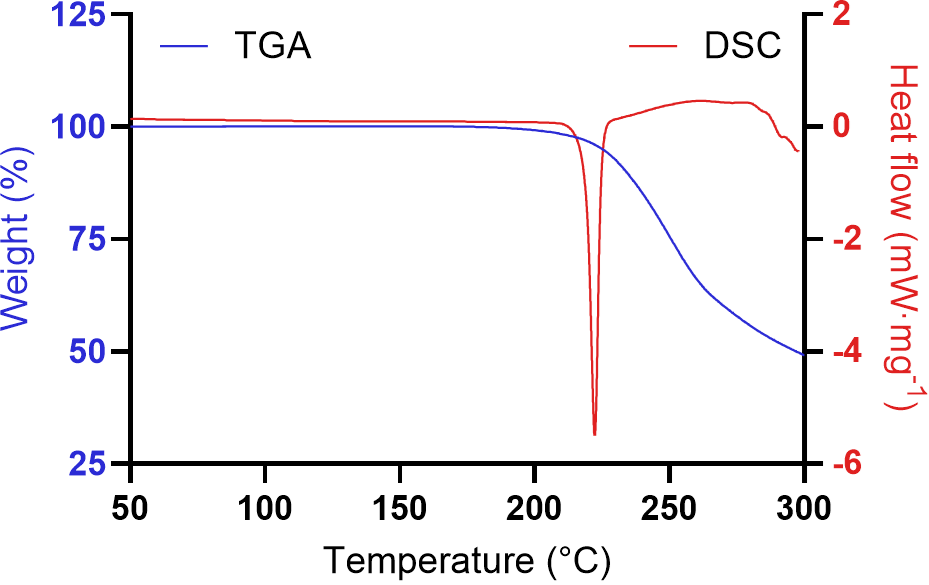
TGA and DSC thermograms of **1·**HCl.

Differential scanning calorimetry (DSC) showed a sharp endothermic peak corresponding to a melting point of 219.0 ± 0.1 °C (with Δ_fus_*H* = 50.4 ± 1.1 kJ/mol) for **1·**HCl (Figure 4), and thermogravimetric analysis (TGA) curves did not show any significant mass loss prior to the melting point, further strengthening the conclusion that **1·**HCl is not present as a hydrate or solvate upon crystallization from EtOH.

### Characterization of 25CN-NBOH (**1**) in solution

**1·**HCl displayed a thermodynamic solubility in water of 8.5 ± 0.3 mg/mL, leading to a solution with a pH of 6.24 ± 0.01. However, the kinetic solubility appears low; when preparing a solution of 5 mg/mL, the container must be shaken for several minutes. Previously, we reported the solubility of **1** in buffer at pH 7.4 to be 0.4 mg/mL, which is sufficient for most biological applications given the high potency of the compound both in vitro and in vivo [6]. Here, we show that if solutions with a higher concentration are needed, pure water could be considered.

According to HPLC analysis, **1·**HCl was found to be stable in solution in phosphate buffer at pH 7.4 at 25 °C, as indicated by the constant peak area (no significant change in AUC) and the absence of new peaks after 4 weeks of storage. In analogy to the buffered solutions, we could not detect additional species besides **1** after 4 weeks in water, when stored at 25 °C. This indicates that aqueous solutions of **1·**HCl may be prepared and stored well in advance of any application or investigation.

**Figure 5:**
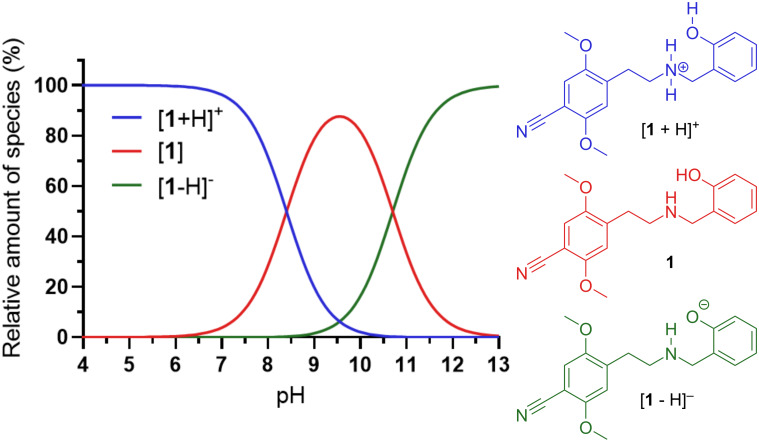
Calculated pH-dependent species distribution curves for 25CN-NBOH (**1**).

Subsequently, the apparent p*K*_a_ values (concentration-based) for **1** were determined to be 8.4 ± 0.1 and 10.7 ± 0.1 for the protonated amine and phenol, respectively, and the calculated pH-dependent species distribution curves for **1** were plotted (Figure 5).

This is reflected by log*D* at different pH values (Table 1). At pH 5, the protonated species dominates, with log*D* = −0.76. At pH 7.4, approximately 90% will be present in the protonated form, alongside 10% of the neutral species (see Figure 5), giving a log*D* of 1.59. However, at pH 9.5, 87% of **1** will be present as the neutral species, resulting in a log*D* value of 2.58.

**Table 1:** Experimentally determined octanol/water distribution coefficient (logD) for 25CN-NBOH (**1**) at different pH values.

pH	5.00	7.40	9.50

log*D*	−0.76 ± 0.01	1.59 ± 0.02	2.58 ± 0.01

In a saturated solution of **1·**HCl in ultrapure water, the pH value was determined to be 6.24, meaning that >99% of **1** will be present in the protonated form, which explains the relatively high solubility.

### Intramolecular hydrogen bonding

Previous pharmacokinetic investigations showed that **1** displays high permeability in the MDCK assay (apical–basal 29 × 10^−6^ cm/s and basal–apical 20 × 10^−6^ cm/s) [6], a well-established in vitro model for assessing blood–brain barrier (BBB) permeability [13]. Moreover, in vivo experiments in rodents showed that maximal brain exposure was rapidly attained and further confirmed that **1** indeed has excellent BBB permeability [6].

Numerous metrics indicate preferred characteristics for central nervous system-targeting compounds, and it is generally accepted that a log*D*_7.4_ around 1–3 is desirable [14]. Thus, the observed log*D*_7.4_ of 1.59 is consistent with the high membrane permeability observed for **1**. This is not necessarily to be expected based on the chemical structure of **1** due to the high intrinsic polarity of the free phenolic moiety [15]. For example, ChemAxon’s calculated clog*D*_7.4_ for 1 is 0.84 – substantially lower than the experimental value. Importantly, due to the proximity of the free secondary amine, it appeared plausible that an intramolecular hydrogen bond (IMHB) could be present under certain conditions.

A molecule dissolved in water must, upon entry into a more hydrophobic environment, such as a lipid bilayer, be able to shed its solvation sphere. This desolvation step can happen more readily when the molecule is able to form an IMHB instead of being strongly coordinated to the solvent. Moreover, an internally hydrogen-bonded conformation is less polar and more rigid in nature, and thus reducing the energetic penalty that is typically associated with this process. The possible impacts of such IMHBs on membrane permeability are well recognized [16–17]. Therefore, it appeared reasonable to speculate that a possible IMHB of **1** could be partially responsible for its high membrane permeability.

To investigate if an IMHB is indeed present in **1**, we combined DFT calculations with NMR spectroscopy titrations. For a system containing an IMHB, it would be expected that replacing the proton (O–H) with a deuteron (O–D) would lead to an isotope effect observable as changes in the ^13^C NMR chemical shift [18–19].

**Figure 6:**
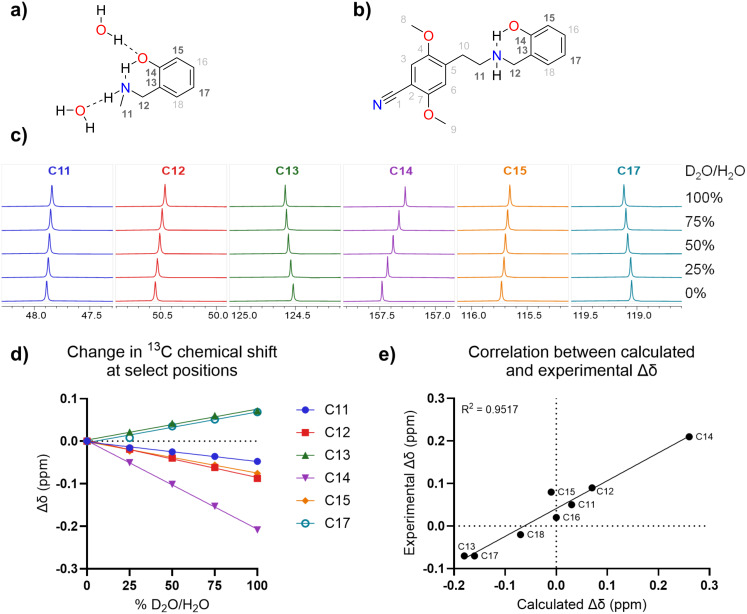
a) Structure of the truncated model compound used for DFT calculations, with explicit water molecules and hydrogen bonds. b) Structure of **1** indicating formation of IMHB, as well as numbering of all carbon positions. c) Overlay of peaks from the ^13^C NMR spectra of **1** with varying percentages of D_2_O/H_2_O in DMSO-*d*_6_. d) Observed changes in the chemical shift. e) Correlation between calculated and experimentally observed chemical shift changes.

DFT calculations were based on a truncated model (Figure 6a) containing the essential elements for intramolecular hydrogen bonding. The structure was considered both in vacuum as well as with DMSO or water as implicit solvents, in the latter two cases with water molecules hydrogen-bonded to the OH and NH groups. The calculated ^13^C chemical shift was compared to the experimentally observed values in Supporting Information File 1. A slightly better correlation between the calculated and experimentally observed chemical shift was obtained for the structure with two water molecules hydrogen-bonded to the OH and NH groups.

The predicted isotope effect was calculated as the difference between the computationally predicted ^13^C chemical shift for the regular model and for models where each O–H and N–H bond was shortened by 0.01 Å (as a proxy for the replacement by deuterium) [20]. Thus, the calculated isotope effect is the sum of the effects of deuteration of the OH and NH positions. However, as isotope effects are transmitted poorly via aliphatic carbon atoms [18], the effect at the phenolic ring can be assumed to stem almost entirely from deuteration of the hydroxy group. The good linear fit (*R*^2^ = 0.95, Figure 6e) indicates that the isotope effects on the chemical shift values could be calculated accurately. Notably, the two-bond deuterium isotope effect on the ^13^C chemical shift reflects the strength of the hydrogen bond [21].

When measuring ^13^C NMR spectra in DMSO-*d*_6_ containing different D_2_O/H_2_O ratios, isotope effects were clearly observed at several positions (Figure 6c and Figure 6d), with a particularly pronounced upfield shift of the phenol *ipso*-carbon atom (C14, Δδ_exp_ = 0.21 ppm). This two-bond isotope effect is a clear indication that the OH group is engaged in an IMHB [19]. Furthermore, the relatively small isotope effect on C11 and C12 also indicates a hydrogen bond to nitrogen (i.e., nitrogen as hydrogen bond acceptor), as the effect caused solely by NH(D) substitution would have been expected to be larger [22]. Since the IMHB in **1** is only slightly weaker than that found in some flavonoids, it is likely strong enough to persist even in aqueous media [23–24].

Based on the measured p*K*_a_ values, at pH 7.4 approximately 10% of **1** is present in the neutral form. Combined with evidence from the isotope effect experiments, this suggests that under physiological conditions, a significant fraction of **1** will be able to adapt a conformation with an IMHB – the species that is most amenable to cross biomembranes. It should be mentioned that there could also be an IMHB present in the protonated form. Although this IMHB may compensate this to some extent, a formal positive charge is nevertheless present, and hence it is more likely that the neutral form with an IMHB is the principal species responsible for the high membrane permeability.

## Conclusion

We optimized the final step of the synthesis of 25CN-NBOH (**1**), providing easier access to this compound. When the hydrochloride salt **1·**HCl was precipitated and recrystallized from ethanol, a single and stable, nonhygroscopic polymorph with a well-defined melting point formed.

A crystal structure determined by SCXRD confirmed the structural assignment, and no water molecules of crystallization or other solvent molecules were incorporated into the crystal packing. XRPD corroborated these findings, indicating a high degree of crystallinity and the presence of a single polymorph in the current work.

Product **1·**HCl was stable in aqueous solution over an extended time (1 month) at 25 °C, with a solubility of 8.5 mg/mL, making it possible to prepare stock solutions for various investigations that can be stored and handled without special precautions.

Compound **1** contains a phenolic moiety, which is typically associated with poor membrane permeability due to the polar nature of this functional group. Based on computationally predicted and experimentally determined secondary deuterium isotope effects, we pinpointed the presence of an IMHB in the neutral form of **1**, wherein the phenol proton interacts with the lone pair on the secondary amine. The p*K*_a_ values of the secondary amine and phenol were found to be 8.4 and 10.7, respectively. At physiological pH, approximately 10% of the relevant neutral species were present in solution. Combined with a log*D*_7.4_ value of 1.59, this was presumably a contributing factor in the favorable membrane permeability of **1**.

Taken together, this investigation further supports the use of 25CN-NBOH (**1**) for investigations into the effects of selective 5-HT_2A_R activation. Our data provides guidance to researchers for the handling and physiochemical properties of the compound when considering its use in various research scenarios.

## Experimental

Reagents and solvents were used as received from commercial vendors, unless otherwise specified. *ortho*-Phosphoric acid (85%, Ph. Eur.), potassium chloride, sodium carbonate (>99.9%), and sodium dihydrogen phosphate monohydrate (Ph. Eur.) were purchased from Merck (Darmstadt, Germany). Acetonitrile (Ph. Eur.), absolute ethanol (Reag. Ph. Eur.), ethyl acetate (HPLC grade), dichloromethane (HPLC grade), ammonia (25%, analytical reagent grade), and diethyl ether (reagent grade) were purchased from VWR (Radnor, PA, USA). Sodium hydrogencitrate sesquihydrate (99%), salicylaldehyde (≥98%), molecular sieves (4 Å, powder, 325 mesh), and hydrogen chloride solution (3 M in methanol, LiChropur™) were purchased from Sigma-Aldrich (St. Louis, MO, USA). 1-Octanol was purchased from Ferak Berlin (Berlin, Germany), triethylamine (99%) from Thermo Fisher Scientific (Fair Lawn, NJ, USA), NaBH_4_ (granulated, >95%) from TCI Europe N.V. (Zwijndrecht, Belgium), and molecular sieves (3 Å, Ø 3–5 mm) from BLD Pharmatech GmbH (Hamburg, Germany). Ultrapure water (18.2 MΩ·cm) was collected from a Purelab Quest (Elga LabWater, High Wycombe, UK). Thin-layer chromatography (TLC) was performed on aluminium sheets precoated with silica gel 60 F_254_ (Merck KGaA, Darmstadt, Germany), and retention factors are denoted *R*_f_. Graphs were produced using GraphPad Prism 10.5, except for NMR spectra, where MestReNova was used. ChemDraw 23.1.2 was used to draw chemical structures.

### Synthesis of 25CN-NBOH·HCl (**1**·HCl)

To a slurry of 2C-CN·HCl [12] (606 mg, 2.50 mmol) in dry EtOH (25.0 mL) were added triethylamine (348 μL, 2.50 mmol, 1.00 equiv), salicylaldehyde (264 μL, 2.50 mmol, 1.00 equiv), and then 4 Å molecular sieves powder (609 mg, 100 wt %), after which the reaction mixture was left to stir under a nitrogen atmosphere for 1.5 hours at room temperature. Subsequently, the reaction was cooled in an ice bath before NaBH_4_ (192 mg, 5.08 mmol, 2.03 equiv) was added in four portions over 15 minutes. The reaction was left to stir at that temperature for an additional 20 minutes before leaving it at room temperature for 35 minutes. Finally, quenching was done by careful addition of aqueous citric acid (12.5 mL, 1 M) while cooling in an ice bath. The resulting mixture was filtered through a pad of Celite and diluted with dichloromethane (75 mL) and water (75 mL), and the aqueous phase was adjusted to pH 8–9 using saturated aqueous Na_2_CO_3_. The layers were separated, the organic phase collected, and the aqueous phase was further extracted with dichloromethane (3 × 25 mL). The combined organic phase was dried over anhydrous Na_2_SO_4_, filtered, evaporated in vacuo, and deposited on Celite. Purification by automated flash column chromatography (Pure C-810 Flash, Büchi Labortechnik GmbH, Essen, Germany) was performed with a normal-phase silica column (25 g FlashPure EcoFlex, Büchi), employing a binary gradient from 0–100% (EtOAc/EtOH 3:1 + 2% aqueous NH_3_)/*n*-heptane. Product fractions were pooled and evaporated in vacuo, providing the product as the free base (679 mg). This was then converted to the HCl salt by dissolving the compound in Et_2_O (10 mL) and adding methanolic HCl (1.1 mL, 3 M, 1.5 equiv). The solids were thrice redissolved in MeOH (10 mL) and evaporated in vacuo, leading to a slightly off-white amorphous solid. Crystallization was done by dissolving the compound in boiling EtOH (32 mL), slow cooling to room temperature, and ensuring complete precipitation by addition of the same volume of Et_2_O and cooling to 0 °C, after which the solids were collected by vacuum filtration. Drying under high vacuum provided the product **1·**HCl as a white solid (643 mg, 1.84 mmol, 74%). *R*_f_ (50% (EtOAc/EtOH 3:1 + 2% aqueous NH_3_)/*n*-heptane) 0.47; ^1^H NMR (600 MHz, DMSO-*d*_6_, δ) 10.24 (bs, 1H), 8.98 (bs, 2H), 7.38 (dd, *J* = 7.5; 1.7 Hz, 1H), 7.35 (s, 1H), 7.24 (td, *J* = 7.7; 1.7 Hz, 1H), 7.14 (s, 1H), 6.96 (dd, *J* = 8.2; 1.2 Hz, 1H), 6.85 (td, *J* = 7.4; 1.1 Hz, 1H), 4.10 (s, 2H), 3.87 (s, 3H), 3.78 (s, 3H), 3.15–3.09 (m, 2H), 3.06–3.00 (m, 2H); ^13^C NMR (151 MHz, DMSO-*d*_6_, δ) 156.01, 155.23, 150.94, 132.97, 131.57, 130.47, 119.06, 118.02, 116.39, 115.36, 114.92, 114.63, 98.51, 56.51, 56.29, 45.45, 45.14, 26.84.

### Recrystallization

Compound **1·HCl** (100 mg) was dissolved in boiling EtOH (5.0 mL) in a 20 mL vial equipped with a magnetic stir bar, resulting in a clear solution. The solution was then allowed to slowly cool to room temperature while stirring, resulting in the formation of a fine white precipitate. After cooling further in the fridge (4 °C) overnight, the solids were collected by vacuum filtration and dried under high vacuum. In this way, approximately 60% of the starting material was recovered as a white solid.

### SCXRD

Crystals of **1·**HCl were grown by slow evaporation of a solution in EtOH at rt. A single crystal suitable for X-ray diffraction studies was mounted and immersed in a stream of nitrogen gas (*T* = 100(1) K). Diffraction data were collected using Mo Kα radiation (λ = 0.71073 Å) with multilayer optics on a Bruker D8 Venture diffractometer. Data collection and cell refinement were performed using the Bruker Apex4 Suite software. Data processing (reduction, merging, and integration) using SAINT and multiscan correction for absorption using SADABS-2016-2 were performed within the Apex4 Suite [25–27]. Mercury 4.0 was used to prepare Figure 2a [28]. The crystal data, data collection, and data processing results are given in Table S1, Supporting Information File 1.

Crystallographic data (fractional atomic coordinates, a list of anisotropic displacement parameters, and a complete list of geometrical data) were deposited in the Cambridge Crystallographic Data Centre (CCDC 2482645).

### XRPD

XRPD was performed with different samples of **1·**HCl and a slurry thereof after equilibration with ultrapure water for 72 h at 25 °C. Samples were measured using an X’Pert PANalytical PRO X-ray diffractometer (PANalytical, Almelo, Netherlands) using Cu Kα radiation (λ = 1.5406 Å), an acceleration voltage of 45 kV, and a current of 40 mV. The samples were measured in reflectance mode in the range of 5–35° 2θ with a step size of 0.013° 2θ and a counting time of 358 s/step. Data was collected using the X’Pert Data Collector software (Version 2.2.4, PANalytical).

### TGA and DSC

The water content was assessed by TGA using a Discovery TGA (TA Instruments, New Castle, DE, USA) with a nitrogen gas flow of 50 mL/min. The sample (5 mg) was heated from 25–300 °C at 10 °C/min. Melting points were determined by DSC using a Discovery DSC 25 (TA Instruments) with a nitrogen gas flow of 50 mL/min. Samples of 2–3 mg were placed in aluminium pans closed with punctured aluminium lids. The samples were heated from 40–300 °C at 10 °C/min, and the melting point was determined as the onset temperature of the peak. The melting point was determined in triplicate. Data was collected and analyzed using the Trios software (V5.1.1 and V5.8.1, TA Instruments).

### Solubility

The solubility of **1·**HCl in water was determined at 25 ± 0.5 °C by adding 30 mg to ultrapure water (3 mL) in screw-capped glass vials, giving a suspension where a solid precipitate could be observed at all timepoints. The samples were ultrasonicated for 3 min before being rotated in a temperature-controlled Incubator Hood TH 30 (Edmund Bühler, Bodelshausen, Germany). Sample aliquots of 600 µL were withdrawn after 30, 48, and 74 h and transferred to Eppendorf tubes. The samples were centrifuged at 19,000*g* for 15 min using a MiniSpin Plus (Eppendorf, Hamburg, Germany) and the supernatant filtered through a 0.45 µm PVDF filter (Ø = 4 mm, Phenomenex) with the first 400 µL being discarded. Samples were diluted with ultrapure water before measurement by a UV spectrophotometry at 241 nm using 10 mm micro quartz cuvettes (Hellma GmbH & Co. KG, Müllheim, Germany) and a Cary 60 UV–vis spectrophotometer (Agilent Technologies). Cary WinUV Simple Reads Application (Version 5.0.0, Agilent Technologies) was used for instrument control and data collection. The experiment was performed in triplicate.

### Stability in solution

The stability of **1** was determined in 67 mM sodium phosphate buffer (pH 7.40, *I* = 0.18 M) and water at 25 ± 0.5 °C. The solutions comprising 0.2 mg/mL of **1·**HCl were kept in a temperature-controlled incubator, and samples were measured by HPLC immediately after sample preparation as well as after one and four weeks for the experiments in phosphate buffer and after four weeks for experiments in ultrapure water. The stability of **1** was assessed based on peak areas. The experiment was performed in triplicate.

### Distribution coefficients

The pH-dependent octanol/buffer distribution coefficients of **1** were determined at 25 ± 0.5 °C using the following buffers: 50 mM sodium citrate (pH 5.00, *I* = 0.15 M), 67 mM sodium phosphate (pH 7.40, *I* = 0.18 M), and 50 mM sodium carbonate (pH 9.50, *I* = 0.15 M). The ionic strength of the buffers was adjusted by adding a calculated amount of potassium chloride. The octanol phases were saturated with the appropriate buffer solution, and vice versa, before the start of the experiment. Solutions of 2, 0.5, and 0.1 mg/mL of the drug in the buffer solutions at pH 5.00, 7.40, and 9.50, respectively, were prepared. The organic phase was hereafter added to the buffer solution in a ratio of 1:9 at pH 5.00 and 7.40 and 1:19 at pH 9.50. The samples were rotated for 24 h in a temperature-controlled incubator at 25 °C. The concentration of **1** in the buffer solution before and after partitioning, *c*_i_ and *c*_w_, respectively, was determined by HPLC, from which distribution coefficients, *D*, were calculated according to


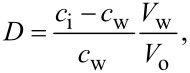


where *V*_w_ and *V*_o_ are the volume of the buffer solution and organic phase, respectively. Each experiment was conducted in triplicate.

### Molar absorption coefficient and p*K*_a_

The molar absorption coefficients and apparent p*K*_a_ values of **1** were determined by spectrophotometry using an inForm instrument from Sirius Analytical (Forest Row, UK). Molar absorption coefficients (absorbance), as a function of the wavelength (185–750 nm) and pH in the interval 2.0–12.0, were determined at 30.8 °C. A stock solution (5.0 mM) of **1·**HCl in *N*-methyl-2-pyrrolidone (NMP) was added to 36 mL of an ionic strength-adjusted buffer solution (*I* = 0.172 M) containing acetate, phosphate, and sodium chloride while stirring at 300 rpm. The concentration of **1** during measurements ranged from 13**–**27 µM (three experiments each with three titrations were performed). Potentiometric titrations from high to low pH and low to high pH were performed by addition of 0.50 M HCl and 0.50 M NaOH, respectively, while UV–vis spectra were recorded using the fiber optic probe (10 mm light path) of the inForm diode array spectrometer. The instrument control and data analysis were performed using the inForm software Version 1.1.3.6 (Sirius Analytical, Forest Row, UK).

### HPLC

Samples from distribution coefficient and stability experiments were analyzed using an Agilent 1260 Infinity system (Agilent Technologies, Santa Clara, CA, USA) equipped with a column oven (Mikrolab Aarhus, Højbjerg, Denmark) and an Agilent 1100 series system (Agilent Technologies), respectively. Reversed-phase chromatography was performed using a Gemini C18 column (150 × 4.60 mm, 5 µm) with a Gemini C18 precolumn (4 × 3.00 mm, Phenomenex, Torrance, CA, USA). Detection was performed at 241 nm, the column temperature was 30 °C, the injection volume was 10 µL, and the flow rate was 1 mL/min. The mobile phase consisted of acetonitrile and 0.1% phosphoric acid in the ratios 35:65 and 25:75, v/v for the distribution coefficient and stability experiments, respectively. The ChemStation software (Version B.04.03, Agilent Technologies) was used for instrument control and data collection.

### Computational chemistry

DFT calculations were performed using the Gaussian 16 software [29]. A truncated model of **1** containing the essential elements for hydrogen bonding was used (see Figure 6a). The structure was geometry-optimized in the gas phase using the B3LYP/6-31G(d,p) level of theory. The effect of the solvent was considered using the PCM approach [30]. To ensure gauge independence, nuclear shielding was calculated using the GIAO approach [31–32].

### NMR spectroscopy

NMR spectra were acquired on a 600 MHz Bruker Avance III HD spectrometer equipped with a 5 mm DCH cryoprobe at 300 K unless otherwise specified. Spectra were processed using MestReNova 15.1.0, automatically phase-corrected, and automatically baseline-corrected (Whittaker smoother). ^1^H and ^13^C NMR chemical shifts were referenced using the appropriate residual solvent as a secondary internal standard (for ^1^H: (DMSO-*d*_6_, δ) 2.50, for ^13^C: (DMSO-*d*_6_, δ) 39.52). Chemical shift (δ) values are reported in ppm, coupling constants (*J*) in Hz, and the peak multiplicity is referred to the following abbreviations: bs (broad singlet), s (singlet), d (doublet), t (triplet), q (quartet), p (pentet), sex (sextet), h (heptet), and m (multiplet).

To investigate the possible presence of an IMHB, water mixtures containing different ratios of deuterated/nondeuterated water (0%, 25%, 50%, 75%, and 100% D_2_O/H_2_O) were prepared, and the free base **1** (15 mg) was dissolved in DMSO-*d*_6_ (2300 μL). Five NMR samples were prepared by thoroughly mixing each water mixture with a portion of the DMSO-*d*_6_ solution in a 1:9 ratio (50 + 450 μL). ^13^C NMR spectra (pulse sequence zgpg30) were acquired at 150.80 MHz with 30° pulses, a spectral width of 36 kHz, collecting 1024 scans with a length of 65536 data points and a relaxation delay of 2.0 s. FIDs were exponentially multiplied with a line broadening factor of 1.0 Hz before Fourier transformation. Spectra were processed with MestReNova.

## Supporting Information

File 1 Further details on SCXRD, deuterium isotope effects, and NMR spectra.

## Data Availability

All data that supports the findings of this study is available in the published article and/or the supporting information of this article.
