# Supplementary material for: Improved synthesis and physicochemical characterization of the selective serotonin 2A receptor agonist 25CN-NBOH
Source: Beilstein J Org Chem. 2026 Jan 22;22:175–84. doi: 10.3762/bjoc.22.11 (PMC12834051; doi:10.3762/bjoc.22.11)
Supplement: File 1 — Further details on SCXRD, deuterium isotope effects, and NMR spectra. [file Beilstein_J_Org_Chem-22-175-s001.pdf]

BEILSTEIN JOURNAL OF ORGANIC CHEMISTRY

Supporting Information

for

Improved synthesis and physicochemical characterization of the selective serotonin 2A receptor agonist 25CN-NBOH

Adrian G. Rossebø, Hannah G. Kolberg, Anders E. Tønder, Louise Kjaerulff, Poul Erik Hansen, Karla A. Frydenvang, Jesper Østergaard and Jesper L. Kristensen

Beilstein J. Org. Chem. **2026,** *22,* 175–184. doi:10.3762/bjoc.22.11

Further details on SCXRD, deuterium isotope effects, and NMR spectra

License and Terms: This is a supporting information file under the terms of the Creative Commons Attribution License (https://creativecommons.org/licenses/by/4.0). Please note that the reuse, redistribution and reproduction in particular requires that the author(s) and source are credited and that individual graphics may be subject to special legal provisions.
The license is subject to the *Beilstein Journal of Organic Chemistry* terms and conditions: (https://www.beilstein-journals.org/bjoc/terms)

Single-crystal X-ray diffraction (SCXRD)

Positions of all nonhydrogen atoms were located by direct methods (SHELXT) [1]. Full-matrix least-squares refinements (SHELXL) [2] were performed on F^2, minimizing $\sum w(F_o^2 - kF_c^2)^2$. The position and anisotropic displacement parameters of the nonhydrogen atoms were refined. Hydrogen atoms were observed in subsequent difference electron density maps, and the hydrogen atoms were included in calculated position with fixed isotropic displacement parameters (U_{iso} = 1.2 U_{eq} for CH, CH_2 and U_{iso} = 1.5 U_{eq} for CH_3), except for hydrogen atoms connected to O or N atoms. For these hydrogen atoms, the position was refined with fixed isotropic displacement parameters (U_{iso} = 1.2 U_{eq} for NH_2; U_{iso} = 1.5 U_{eq} for OH).

Refinement (228 parameters, 4864 unique reflections) converged at R_F = 0.0401, wR_F^2 = 0.0933 (4238 reflections with $F_o > 4\,\sigma(F_o)$, $w^{-1} = (\sigma^2(F_o^2) + (0.0379\,P)^2 + 0.3263\,P)$, where $P = (F_o^2 + 2\,F_c^2)/3$, S = 1.042). The residual electron density varied between −0.29 and 0.41 e·Å^{-3}. Complex scattering factors for neutral atoms were taken from International Tables for Crystallography [3] as incorporated in SHELXL. The structure refinement details are summarized in the table below.

Table S1: SCXRD crystal data, data collection, and refinement data for **1·HCl**.

Formula	$C_{18}H_{20}N_2O_3$; HCl
Mw (g/mol)	348.82
Temperature (K)	100(1)
Crystal class	triclinic
Space group	*P*-1
Cell parameters	a = 7.9797(9) Å<br>b = 9.0829(12) Å<br>c = 12.723(2) Å<br>α = 102.530(6)°<br>β = 101.278(5)°<br>γ = 97.587(5)°
V (Å^3), Z	868.0(2) / 2
F(000)	368
d_{calc} (g/cm^3)	1.335
λ	0.71073 Å (Mo*K*α)
θ_{max} (°)	2.34 < θ < 29.58
Reflections total/unique	37669/4864
R_{merge}	0.0416
No. parameters/restraints	228/0
Reflections ($I > 2\,\sigma(I)$)	4238
R1/wR2 ($I > 2\,\sigma(I)$)	0.0338/0.0877
R1/wR2 (all reflections)	0.0401/0.0933
Goodness-of-fit on F^2	1.042
ρ_{max} / ρ_{min} (eÅ^{-3})	0.41 / -0.29

Secondary deuterium isotope effects

Predicted nuclear shielding and experimental chemical shift

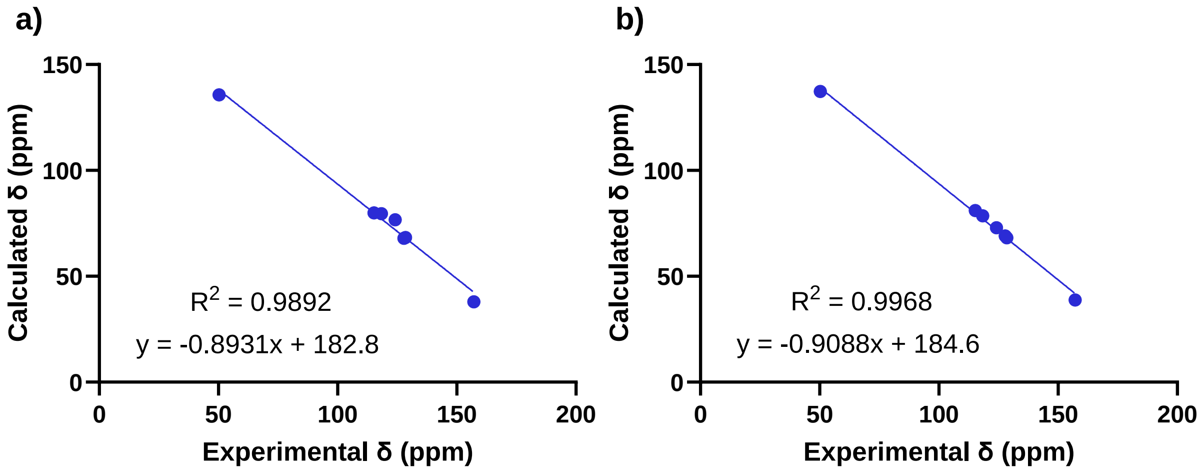

Figure S1: Plot of calculated ^{13}C nuclear shielding vs experimental chemical shift. Calculated values a) in vacuum and b) with water molecules hydrogen-bonded and as implicit solvent. Although the difference is small, the fit is better in b), and the model is more physically correct.

Predicted and experimental chemical shift changes

Table S2: Predicted and experimental deuterium isotope effects on the ^{13}C chemical shift (in ppm). a) The OH hydrogen atom is simulated as deuterium by shortening the O–H bond length. b) The NH hydrogen atom is simulated as deuterium by shortening the N–H bond length. c) Sum of $\Delta\delta_{calc}$ (OH) and $\Delta\delta_{calc}$ (NH).

Carbon no.	$\Delta\delta_{calc}$(OH) [a]	$\Delta\delta_{calc}$(NH) [b]	$\Delta\delta_{calc}$ [c]	$\Delta\delta_{exp}$
**C11**	-0.09	0.12	0.03	0.05
**C12**	-0.05	0.12	0.07	0.09
**C13**	-0.21	0.03	-0.18	-0.07
**C14**	0.25	0.01	0.26	0.21
**C15**	-0.01	0	-0.01	0.08
**C16**	-0.01	-0.01	-0.02	0.02
**C17**	-0.15	-0.01	-0.16	-0.07
**C18**	-0.06	-0.01	-0.07	-0.02

NMR spectra

1·HCl: ^{1}H NMR (600 MHz) and ^{13}C NMR (151 MHz) in DMSO-d_6

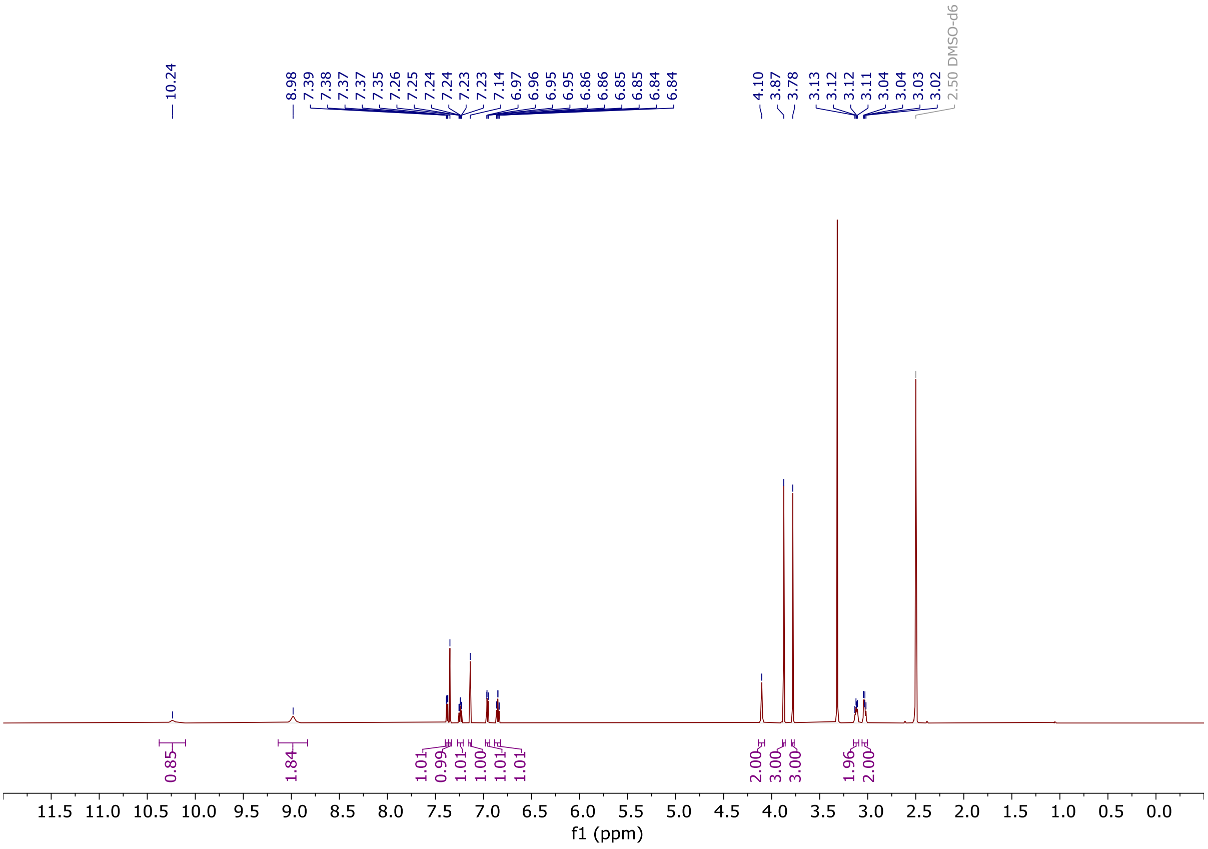

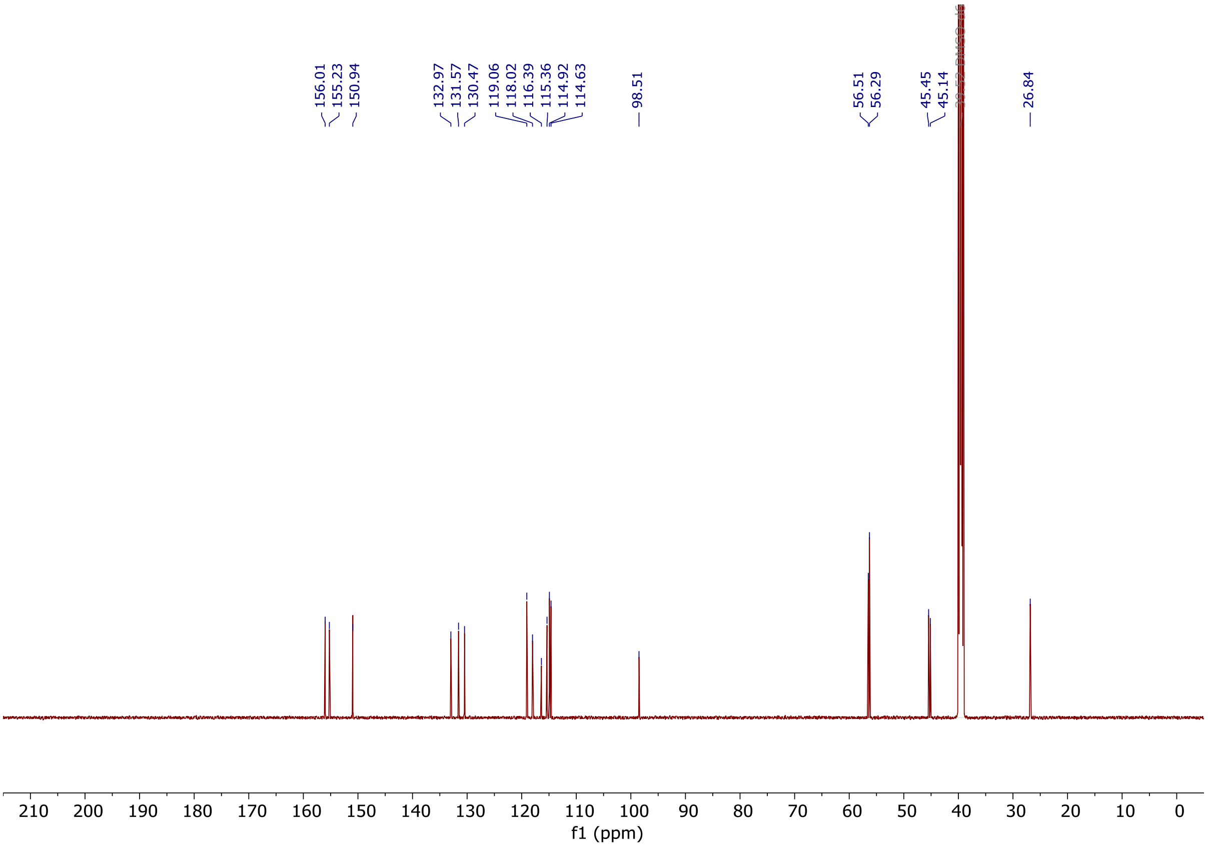

References

1 G. M. Sheldrick, SHELXT – Integrated space-group and crystal-structure determination, *Acta Crystallogr. Sect. Found. Adv.*, 2015, **71**, 3–8.

2 G. M. Sheldrick, Crystal structure refinement with SHELXL, *Acta Crystallogr. Sect. C Struct. Chem.*, 2015, **71**, 3–8.

3 A. J. C. Wilson, *International Tables for Crystallography*, Kluwer Academic Publishers, Dordrecht, The Netherlands, 2006, vol. C.
